# Prioritization of patients for surgery in Canada: The case of hip and knee replacement surgeries in Newfoundland

**DOI:** 10.1002/puh2.104

**Published:** 2023-07-12

**Authors:** Anh Thu Vo, Yanqing Yi, Maria Mathews, James Valcour, Michelle Alexander, Marcel Billard

**Affiliations:** ^1^ Faculty of Medicine Memorial University of Newfoundland, St. John's Newfoundland Canada; ^2^ Schulich School of Medicine & Dentistry Western University London Ontario Canada; ^3^ Clinical Efficiency Program Eastern Health, St. John's Newfoundland Canada; ^4^ Central Intake Division Clinical Efficiency Program Eastern Health, St. John's Newfoundland Canada

**Keywords:** Canada, hip replacement surgery, knee replacement surgery, logistic regression, Newfoundland, patients, priority level, single‐entry model, wait time

## Abstract

**Background:**

Single‐entry models (SEM) improve wait times for hip and knee replacement, but little is known whether prioritization implemented in SEM can help meet the benchmarks for consolation/surgery. This study aimed to determine the impact of prioritization on receiving consultation and surgery within the benchmarks.

**Methods:**

This is a retrospective cohort study for which two administration databases were linked. Logistic regression was used to investigate the impact of prioritization on receiving consultations and surgery within the benchmarks of 90 and 182 days, respectively, adjusting for patients’ characteristics and preference for surgeon.

**Results:**

1,967 patients were included in this study. The odds ratios of having consultation within 90 days for hip replacement patients in priorities 1 and 2 (high priority) were 57.24 (CI: 23.16–141.47) and 14.63 (CI: 6.44–33.25), respectively, compared with those in priority 3. For knee replacement, patients with higher priority were more likely to have consultation within 90 days. Although priority levels were not related to having surgery within 182 days for knee replacement, hip replacement patients with priority 1 (CI: 0.2–0.75) and 2 (CI: 0.16–0.54) were less likely to have surgery within 182 days, compared with those with priority 3.

**Conclusion:**

Patients with high priority levels were more likely to have consultation within 90 days for hip and knee replacements. SEM may not help have surgery within 182 days. Prioritization has no impact on receiving surgery within 182 days for knee replacement, but hip replacement patients with high priority were less likely to have surgery within 182 days.

## INTRODUCTION

Long wait times for hip and knee replacement surgeries have been a significant issue across Canada [1, 2, 3]. Patients waiting over 12 months for arthroplasty surgery have progressive pain, disability, and worsen health‐related quality of life [4, 5, 6]. To reduce the long wait times and its impact on patients’ health, Canada's first ministers agreed to adopt the national benchmark of 182 days for total hip and knee replacement surgery in 2005 [7]. The National Standards Committee of the Canadian Orthopaedic Association (COA) recommended that the maximum acceptable wait time be within 90 and 182 days, respectively, for consultation and surgery [8]. Most Canadian health jurisdictions have developed innovations to meet these benchmark targets, one of which is single‐entry models (SEM).

SEM manages referrals through a central intake system, in which patients can see the next available surgeon based on priority at triage [9, 10, 11]. SEM helped improve referral and triage systems thus reducing wait time for hip and knee surgeries [12, 13, 14, 15, 16, 17, 18, 19]. In Canada, several provinces adopted SEM, including Alberta [13, 14], British Columbia [15], Ontario [16], Manitoba [11, 17], and Nova Scotia [18]. Newfoundland and Labrador (NL) was ranked at the bottom of performances among Canadian provinces in achieving the 90 and 182 days benchmarks, respectively, for consultation and surgery for hip and knee replacements before implementing SEM. In 2010, the NL provincial government initiated the Orthopedic Central Intake program (OCI) in the Eastern Health Region, the largest health authority in NL, which serves on about 60% of the NL population. The main role of the central intake model is to improve the flow of referrals and allows patients to see the next available specialist. Our recent results using the same data [19] revealed that patients with higher urgent levels had consultation booked earlier thus shorter total wait time for knee and hip replacement surgeries. However, it is unknown whether the magnitude of reduced wait times is large enough to achieve the benchmarks for consultation and surgery with prioritization implemented in SEM.

Factors, including age [20, 21], patient preference for surgeons [22, 23], priority levels [22], and incomplete referral forms [22, 24], might impact wait times for consultation and surgery. The study in Ref. [25] claimed that prioritization could make patients with low priority never reach the top of the waiting list when many higher urgent patients are booked with surgeons. It is speculated that the influence of priority levels on meeting the benchmarks of wait times might be different when other factors weight in. In addition, a downward trend in a percentage of patients receiving surgery within 182 days was reported in Canada [22, 26]. A similar trend was also observed in NL. The percentage of patients receiving surgery within 182 days decreased from 95% in 2014 to 65% in 2019 for hip replacement and 92% in 2014 to 65% in 2019 for knee replacement in NL [22].

After accounting for the effect of other factors, does prioritization help meet the established benchmarks for consultation and surgery? The aim of this research was to study the association between priority levels and receiving consultation and surgery within the benchmarks of 90 and 182 days, respectively, adjusting for patients’ age, diagnostic type, preference for surgeon, and time of referral.

## METHODS

### Study design, setting, and population

This is a retrospective cohort study of patients with orthopedic consultation booked through OCI and having surgery between 2011 and 2019 in Newfoundland, Canada. The study population is patients who age 18 or older and are referred to the OCI clinic for hip or knee replacement surgeries. The study cohort was identified through linking the two administration databases, the OCI database to the Total Joint Assessment Centre (TJAC) database at the OCI clinic of Eastern Health Region of NL. The follow‐up was from referral received to having surgery.

### Data sources

Dates of having consultation and surgery were extracted from the OCI and the TJAC databases. The OCI database recorded the information on the referral form, including primary affected joints (hip or knee), diagnosis type, patient's preference for a specific surgeon, referral form status, and the date of referral. The OCI database also included priority levels (P1‐the high priority routine, P2‐moderate, P3‐low vs. P4‐probably unsuitable for surgery) assigned by the OCI clinic, the date of consultation booked, and the name of surgeon. The TJAC database contained the date of decision made to treat, date for surgery received, and the name of the surgeon. Both OCI and TJAC databases include the NL health care plan numbers of patients, which was used to link the two databases. Of the total 25,635 referrals to OCI from 2011 to 2019 for primary hip/knee arthroplasty, patients without having surgeries (*n* = 20,478) and patients under age 18 or having multiple linkages or other linkage error were excluded. A total of 1967 patients with a unique linkage and matched surgeons were identified and included in the analysis. More details on the linkage of the two databases can be found in our previous study [19].

### Study variables

The study outcome variables were receiving hip and knee replacement consultation and surgery within established benchmarks (yes/no), that is, receiving consultation within 90 days of initial referral and receiving surgery within 182 days after decision to treat was made. Wait time for consultation was calculated based on dates of referral and first consultation, and wait time for surgery based on dates of decision to treat and surgery received. Those dates were extracted from the OCI and TJAC databases, and the calculations of wait times for consultation and surgery are based on the COA's definition [8].

The values of the independent variables, diagnosis (osteoarthritis vs. others), patient's preference (next available surgeon vs. specific surgeon), initial referral status (incomplete vs. completed), and priority levels were extracted and/or formatted based on the information from the OCI and TJAC databases. Birth year of a patient was extracted from the information contained in the health‐care plan number to calculate age at referral, which was categorized as <65 versus ≥65 in the analysis. To account for the time trend in a percentage of patients meeting the benchmarks, year of referral was grouped into three periods, 2011–2013, 2014–2016, and 2017–2019.

### Data analysis

For hip and knee replacement patients, summary statistics, including numbers and percentages of cases receiving consultation within 90 days and receiving surgery within 182 days, were reported for age group, diagnosis, priority level, initial referral form status, patient's preference for the surgeon, and year of referral. The chi‐square test or Fisher's exact test was performed to explore the association between having a consultation or surgery within the benchmarks and the independent variables.

Logistic regression models were used to evaluate the association between receiving consultation within 90 days (yes/no) and receiving surgery within 182 days (yes/no) and priority levels, while adjusting for other factors in the model. The direct approach [27] was used to examine the potential factors that may impact receiving consultation and surgery within the benchmarks. The multiple logistic regression model included age group, diagnosis type, patient's preference, initial referral status, year of referral, and priority level as independent variables for hip replacement and had an additional interaction of patient's preference by year of referral to account for the interaction effect and the interaction of patient's preference by priority level for the analysis for knee replacement. The multicollinearity assumption was examined using the Pearson correlation matrix and the value of variance inflation factor. The criterion of correlation less than 0.8 and variance inflation factor smaller than 10 was used to conclude lack of multicollinearity [28]. The Hosmer–Lemeshow test was used to check the goodness of fit of the models.

### Ethical considerations

Newfoundland and Labrador Health Research Ethics Board (HERB) and Eastern Health Process for Review and Approval of Research approved the study. Eastern Health provided the deidentified data for analysis.

## RESULTS

There were a total of 808 patients with hip replacement and 1159 patients with knee replacement. Tables 1 and 2 summarize the characteristics of patients receiving consultation and surgery for hip replacement and knee replacement, respectively. Tables 3 and 4 present the results on effects of factors on the probability of meeting the benchmarks, respectively, for consultation and surgery, where priority 4 (*n* = 2) for hip replacement and (*n* = 6) for knee replacement were excluded from the logistic regression analysis because of the small sample sizes.

**TABLE 1:** The characteristics and the proportion in meeting the benchmarks of wait times for consultation and surgery of hip replacement patients in Eastern Health of Newfoundland, Canada from 2011 to 2019^a^.

Variables	*n* (%)	Wait time for consultation			Wait time for surgery		
		≤90 days (*n*, %)	>90 days (*n*, %)	*p* value^e^	≤182 days (*n*, %)	>182 days (*n*, %)	*p* value^e^
**Age group (years)**				0.010			0.612
<65	363 (45)	219 (60)	144 (40)		212 (58)	151 (42)	
≥65	445 (55)	307 (69)	138 (31)		252 (57)	193 (43)	
**Diagnosis** **^b^**				0.660			0.108
Osteoarthritis	716 (89)	468 (65	248 (35)		404 (56)	312 (44)	
Others	92 (11)	58 (63)	34 (37)		60 (65)	32 (35)	
**Priority**				<0.0001			<0.0001
P1	195 (24)	162 (83)	33 (17)		123 (63)	72 (37)	
P2	537 (66)	357 (66)	180 (34)		281 (52)	256 (48)	
P3	74 (9)	7 (9)	67 (91)		60 (81)	14 (19)	
P4	2 (1)	0 (0)	2 (100)		0 (0)	2 (100)	
**Initial referral** **^c^**				0.010			0.616
Incomplete	32 (4)	14 (44)	18 (56)		17 (53)	15 (47)	
Complete	776 (96)	512 (66)	264 (34)		447 (58)	329 (42)	
**Patient's preference**				0.400			0.024
Next available	582 (72)	384 (66)	198 (34)		320 (55)	262 (45)	
Specific surgeon	226 (28)	142 (63)	84 (37)		144 (64)	82 (36)	
**Year of referral** **^d^**				<0.0001			<0.0001
2011–2013	239 (30)	104 (44)	135 (56)		165 (69)	74 (31)	
2014–2016	306 (38)	212 (69)	94 (31)		137 (45)	169 (55)	
2017–2019	263 (32)	210 (80)	53 (20)		162 (62)	101 (38)	

Abbreviation: OCI, Orthopedic Central Intake.

^a^ Sample size *n* = 808.

^b^ A total hip replacement surgery for osteoarthritis or other hip arthritis disorders.

^c^ A standard referral form status when it was first sent to the OCI by family doctors.

^d^ The year when a patient was referred to the OCI for hip replacement assessment.

^e^ From the chi‐square test or Fisher's exact test.

**TABLE 2:** The characteristics and the proportion in meeting the benchmarks of wait times for consultation and surgery of knee replacement patients in Eastern Health of Newfoundland, Canada from 2011 to 2019^a^.

Variable	*n* (%)	Wait time for consultation (WT1)			Wait time for surgery (WT2)		
		≤90 days (*n*, %)	>90 days (*n*, %)	*p* value^e^	≤182 days (*n*, %)	>182 days (*n*, %)	*p* value^e^
**Age group (years)**				0.006			0.123
<65	560 (48)	244 (44)	316 (56)		231 (41	329 (58	
≥65	599 (52)	309 (52)	290 (48)		274 (46)	325 (54)	
**Diagnosis** **^b^**				0.196			0.163
Osteoarthritis	1027 (89)	497 (48)	530 (52)		440 (43)	587 (57)	
Others	132 (11)	56 (42)	76 (58)		65 (49)	67 (51)	
**Priority**				<0.0001			0.271
P1	116 (10)	85 (73)	31 (27)		59 (51)	57 (49)	
P2	754 (65)	457 (60)	297 (40)		315 (42)	439 (58)	
P3	283 (24)	11 (4)	272 (96)		128 (45)	155 (55)	
P4	6 (1)	0 (0)	6 (100)		3 (50)	3 (50)	
**Initial referral** **^c^**				0.018			0.015
Incomplete	68 (6)	23 (34)	45 (66)		20 (29)	48 (71)	
Complete	1091 (94)	530 (49)	561 (51)		485 (44)	606 (56)	
**Patient's preference**				0.732			0.008
Next available	776 (67)	373 (48)	403 (52)		317 (41)	459 (59)	
Specific surgeon	383 (33)	180 (47)	203 (53)		188 (49)	195 (51)	
**Year of referral** **^d^**				<0.0001			<0.0001
2011–2013	396 (34)	113 (29)	283 (71)		198 (50)	198 (50)	
2014–2016	473 (41)	256 (54)	217 (46)		154 (33)	319 (67)	
2017–2019	290 (25)	184 (63)	106 (37)		153 (53)	137 (47)	

Abbreviation: OCI, Orthopedic Central Intake.

^a^ Sample size *n* = 1159.

^b^ A total knee replacement surgery for osteoarthritis or other knee arthritis disorders.

^c^ A standard referral form status when it was first sent to the OCI by family doctors.

^d^ The year when a patient was referred to the OCI for knee replacement assessment.

^e^ From the chi‐square test or Fisher's exact test.

**TABLE 3:** The predictors of receiving consultation within 90 days among hip or knee replacement patients in Eastern Health of Newfoundland, Canada from 2011 to 2019.

Variables	Adjusted ORs	95% CI	*p* value
Hip replacement surgery consultation^a^			
Age group (years)			0.523
<65 vs. ≥65	0.9	0.6–1.3	
Diagnosis			0.752
Osteoarthritis vs. others	0.9	0.5–1.6	
Patient's preference			0.159
The next available vs. specific surgeon	1.3	0.9–1.9	
Initial referral			0.095
Incomplete vs. complete	0.5	0.2–1.1	
Priority			<0.0001
P1 vs. P3	57.2	23.1–141.5	
P2 vs. P3	14.6	6.4–33.3	

Knee replacement surgery consultation^b^			
Age group			0.464
<65 vs. ≥65	0.9	0.7–1.2	
Diagnosis			0.024
Osteoarthritis vs. others	0.6	0.3–0.9	
Patient's preference × year of referral			0.001
(The next available vs. specific surgeon)^c^	0.8	0.2–3.0	
Priority × patient's preference			0.005
(P1 vs. P3) at specific surgeon	45.1	14.8–137.6	
(P2 vs. P3) at specific surgeon	9.3	3.5–24.8	
(P1 vs. P3) at the next available	106.4	38.0–298.0	
(P2 vs. P3) at the next available	58.4	25.1–135.9	
Initial referral			0.146
Incomplete vs. complete	0.6	0.3–1.2	

^a^ Multiple regression model for *hip replacement consultation*, including age group, diagnosis, patient's preference for surgeon, initial referral, year of referral, and priority level.

^b^ Multiple regression model for *knee replacement consultation*, including age group, diagnosis, age group, diagnosis, patient's preference for surgeon, initial referral, year of referral, priority level, patient's preference × year of referral, and patient's preference × priority.

^c^ OR at P3 and year of referral (2017–2019) and other ORs will be provided upon request.

**TABLE 4:** The predictors of receiving surgery within 182 days among hip or knee replacement patients of Eastern Health of Newfoundland, Canada from 2011 to 2019.

Variables	Adjusted ORs	95% CI	*p* value
Hip replacement surgery^a^			
Age group (years)			0.786
<65 vs. ≥65	1.0	0.8–1.4	
Diagnosis			0.517
Osteoarthritis vs. others	0.9	0.5–1.4	
Patient's preference			0.011
The next available vs. specific surgeon	0.7	0.5–0.9	
Priority			0.0004
P1 vs. P3	0.4	0.2–0.7	
P2 vs. P3	0.3	0.2–0.5	

Knee replacement surgery^b^			
Age group			0.152
<65 vs. ≥65	0.8	0.7–1.1	
Diagnosis			0.137
Osteoarthritis vs. others	0.7	0.5–1.1	
Patient's preference × year of referral			0.003
(The next available vs. specific surgeon)^c^	1.3	0.8–2.1	
Priority			0.445
P1 vs. P3	1.1	0.7–1.7	
P2 vs. P3	0.9	0.6–1.2	

^a^ Multiple regression model for *hip replacement surgery*, including age group, diagnosis, patient's preference for surgeon, year of referral, and priority level.

^b^ Multiple regression model for *knee replacement surgery*, including age group, diagnosis, age group, diagnosis, patient's preference for surgeon, initial referral, year of referral, priority level, and patient's preference × year of referral.

^c^ OR at 2017–2019 and other ORs will be provided upon request.

For consultation within the benchmark of 90 days, age, priority levels, initial referral form completed or not, and year of referral were found to be significantly related to receiving consultation within the benchmark for both hip and knee replacements in the univariate analysis (Tables 1 and 2). In the multiple logistic regressions, age and initial referral became insignificant after adjusting for other covariates. The effect of priority levels differed by patient's preference for knee replacement, adjusting for other factors and the interaction of patient's preference by year of referral (Table 3). With hip replacement surgery, 83%, 66%, and 9% of patients in priorities 1, 2, and 3, respectively, received consultation within 90 days of referral (Table 1). A patient assigned priority 1 or 2 was more likely to have consultation within 90 days than a patient with priority 3 (Table 3).

For knee replacement, the proportions of patients receiving consultation within 90 days were 73%, 60%, and 4% for priorities 1, 2, and 3, respectively (Table 1). Patients with priorities 1 and 2 were more likely to have consultation within 90 days than those with priority 3 for both of choosing the next available surgeon and requesting a specific surgeon. The odds of having consultation within 90 days for patients in priorities 1 and 2 were 106.4 and 58.4 times, respectively, of that for patients in priority 3 when choosing the next available surgeon. For those who requested a specific surgeon, the OR was 45.1 and 9.3 for patients in for priorities 1 and 2, respectively, when compared with those in priority 3 (Table 3). A patient with osteoarthritis was less likely to have consultation than a patient with other arthritis disorders among patients with knee replacement, whereas diagnosis was insignificant for hip replacement (Table 3).

For receiving surgery within the benchmark of 182 days, Table 4 reports the effects of factors on meeting the benchmark from the multiple logistic regressions. Age and diagnosis remained insignificant for both hip and knee surgeries in the logistic regressions, the same as that observed in the univariate analysis (Table 1). For hip replacement surgery, 55% of patients who chose the next available surgeon had surgery within 182 days, whereas 64% received surgery among those preferred specific surgeon (Table 1). Patients choosing the next available surgeon for consultation were less likely to have surgery within 182 days than patients requesting a specific surgeon (Table 4). Overall, 81% patients in priority 3 had surgery within the benchmark but 63% and 52% for priorities 1 and 2, respectively (Table 1). Patients with high priority were less likely to have surgery within 182 days than those in low priority. Odds of having surgery within 182 days for patients in priorities 1 and 2 were 0.4 and 0.3, respectively, when compared with those in priority 3 (Table 4). For knee replacement surgery, the interaction between patient's preference and year of referral was significant. Priority was not significantly related to the likelihood of having surgery within 182 days after adjusting for age, diagnosis, patient's preference, year of referral, and the interactions.

## DISCUSSION

This study found significant effects of priority levels on meeting the benchmark for hip or knee replacement consultation but not for having surgery, after adjusting for diagnosis type, patient's preference and age, and year of referral as well as possible interactions.

For having consultation within 90 days of referral, patients with priorities 1 and 2 were more likely to receive consultation within the benchmark in comparison with those with priority 3 for both hip and knee replacements. This result aligns with the evidence reported in the studies [24, 29] that prioritization through the SEM could allow patients with higher urgent to have short wait times for consultation, surgery, or a total wait times than their counterparts. Moreover, wait‐time benchmarks allow to improve access to care and provide a greater health system accountability and transparency [8]. Our findings suggest that prioritization improved timely access to orthopedic surgeons for consultation within the benchmark of 90 days.

This study also revealed that priority levels were not significantly associated with having knee replacement surgery within the benchmark of 182 days, whereas hip replacement patients with high priority (priority 1 or 2) were less likely to be scheduled for surgery within 182 days than patients with low priority (priority 3). Urgent clinical ratings for hip and knee replacement surgeries depend on criteria, including pain, stiffness, function, and others [25, 30] determined by orthopedic surgeons. A study conducted in Manitoba reported that worse Oxford‐12 score was related to receiving surgery for hip and knee replacements within the benchmark, and BMI was significant for hip replacement surgery [11]. Further research is needed to rescreen the referral forms of the patients with hip replacement surgery who were assigned priority 3 and to investigate why those patients were more likely to have surgery within 182 days than patients with higher priorities.

As for the adjusting factors, this study found that a patient requiring knee replacement surgery due to osteoarthritis was less likely to receive consultation within 90 days than a patient needing knee replacement surgery due to other knee arthritis disorders. We did not find any association between diagnosis type and receiving consultation within 90 days for hip replacement. Regarding patient's preference, choosing the next available surgeon may allow patients to see a surgeon for consultation sooner [30]. Our previous study [19] analyzed the same data using the Cox regression [31], which reported that the patients choosing the next surgeon were more likely to book a consultation sooner than those requesting a specific surgeon. This study did not find any association between patient's preference and receiving consultation within the benchmark of 90 days in hip replacement surgery. It seems that the reduced wait time reported from our previous study was not sufficiently large such that the probability of meeting the 90‐day benchmark among patients who chose the next available surgeon is higher than that of patients who preferred a specific surgeon.

This study also found that choosing the next available surgeon for consultation led to less likely having a hip replacement surgery within 182 days compared with requesting a specific surgeon. Health providers and resource availability are important factors on how quickly patients have a total joint replacement surgery. The unavailability of human resources in the operation room or inpatient hospital beds may result in delayed elective surgery [32]. Patient factors, such as readiness for surgery, comorbidity, and gender, may also influence wait times between consultation with a surgeon and the date of surgery. Further research is needed to explore these factors and incorporate them to produce a more complete assessment of effects of prioritization on wait times.

### Limitations

Although this study revealed that prioritization implemented in SEM helped have consultation within the benchmark of 90 days for patients with high priority levels thus might improve the referral and triage system, there are several limitations. First, the sample data was from the largest health authority region of NL, which might not be representative of other health authority regions in NL. The strength of evidence might differ when generalized to other health authority regions or provinces. Second, the sample only included patients with one to one match when linking the OCI and TJAC databases, and those with multiple linkages were excluded. As a result, the study may underestimate the effects of prioritization on receiving consultation and surgery within the established benchmarks. However, using unique link guarantees the accuracy in extracting wait times. Third, there are many patients’ and contextual factors, including clinical comorbidities and gender that are not available in the databases. Further studies are needed to consider the influence of these factors to assess the effect of prioritization on having consultation and surgery within the established benchmarks.

## CONCLUSION

SEM is seen as a promising tool to reduce overall wait times for surgery. Our analyses showed that prioritization implemented in SEM increased the likelihood that patients with high priority (priorities 1 and 2) have consultation within 90 days. However, prioritization may not help reach the goal of having surgery within the benchmark of 182 days. Hip replacement patients with high priority (priorities 1 and 2) were less likely to receive surgery within 182 days. Choosing the next available surgeon also influenced the likelihood of having a consultation within 90 days, but its influence on receiving surgery within 182 days was significant in a reverse way for hip replacement. Further studies are needed to consider additional patient factors and availability of resources to explore the role of prioritization on receiving consultation and surgery within established benchmarks in SEM.

## AUTHOR CONTRIBUTIONS

Anh Thu Vo contributed to ethical submission, data analysis, and interpretation of the data and crafted the manuscript. Yanqing Yi contributed to rewriting the sections of introduction, method, and results of the revised manuscript. Yanqing Yi, Maria Mathews, and James Valcour contributed to study design and supervised study analysis, interpretation, and writing. Marcel Billard and Michelle Alexander contributed to study design and supported linkage data and ethical submission. All authors read and approved the final manuscript.

## CONFLICT OF INTEREST STATEMENT

No conflict of interest to report.

## FUNDING INFORMATION

No funding to report.

## Data Availability

The data sets used and/or analyzed during the current study are available from the corresponding author on reasonable request and upon approval of Easter Health, Newfoundland, and Labrador (rpac@easternhealth.ca).
